# Anticancer Mechanisms of Bioactive Compounds from Solanaceae: An Update

**DOI:** 10.3390/cancers13194989

**Published:** 2021-10-05

**Authors:** David O. Nkwe, Bonolo Lotshwao, Gaolathe Rantong, James Matshwele, Tebogo E. Kwape, Kabo Masisi, Goabaone Gaobotse, Kathleen Hefferon, Abdullah Makhzoum

**Affiliations:** 1 Department of Biological Sciences and Biotechnology, Botswana International University of Science and Technology, Palapye, Botswana; bonolo.lotshwao@studentmail.biust.ac.bw (B.L.); rantongg@biust.ac.bw (G.R.); kwapet@biust.ac.bw (T.E.K.); masisik@biust.ac.bw (K.M.); gaobotseg@biust.ac.bw (G.G.); 2 Department of Chemical and Forensic Sciences, Botswana International University of Science and Technology, Palapye, Botswana; james.matshwele@studentmail.biust.ac.bw; 3 Department of Applied Sciences, Botho University, Gaborone, Botswana; 4 Virology Laboratory, Department of Cell & Systems Biology, University of Toronto, Toronto, ON M5S 3B2, Canada; kathleen.hefferon@alumni.utoronto.ca

**Keywords:** Solanaceae, cancer, anticancer/antitumour, apoptosis, cell cycle, transcription, autophagy, signalling

## Abstract

**Simple Summary:**

The Solanaceae plant family has been a good source of natural compounds that can treat several diseases, including cancer. However, the site and mechanism of action of these compounds in cells has not been entirely clear. With ongoing developments in biological science research, it has become possible to study and identify some of the cellular targets. In this review, we assess publications from the last five years and identify research articles that explain how some of these compounds may work against cancer. These studies show that a number of different components or pathways in cells are targeted by these compounds to inhibit cell proliferation. Interestingly, some compounds have multiple targets and may be effective against different types of cancer. This knowledge may allow scientists to design new and more effective anticancer drugs.

**Abstract:**

Plants continue to provide unlimited pharmacologically active compounds that can treat various illnesses, including cancer. The Solanaceae family, besides providing economically important food plants, such as potatoes and tomatoes, has been exploited extensively in folk medicine, as it provides an array of bioactive compounds. Many studies have demonstrated the anticancer potency of some of the compounds, but the corresponding molecular targets are not well defined. However, advances in molecular cell biology and in silico modelling have made it possible to dissect some of the underlying mechanisms. By reviewing the literature over the last five years, we provide an update on anticancer mechanisms associated with phytochemicals isolated from species in the Solanaceae plant family. These mechanisms are conveniently grouped into cell cycle arrest, transcription regulation, modulation of autophagy, inhibition of signalling pathways, suppression of metabolic enzymes, and membrane disruption. The majority of the bioactive compounds exert their antiproliferative effects by inhibiting diverse signalling pathways, as well as arresting the cell cycle. Furthermore, some of the phytochemicals are effective against more than one cancer type. Therefore, understanding these mechanisms provides paths for future formulation of novel anticancer drugs, as well as highlighting potential areas of research.

## 1. Introduction

Globally, cancer is the leading cause of death, with growing incidence and mortality burdens [1]. Genetic mutations are common causes of most cancers, although an understanding of epigenetic factors in cancer pathology is also gaining ground. These mutations allow cells to evade apoptosis and proliferate uncontrollably. Some of these cancer cells may acquire the ability to migrate from the initial primary site to other distant tissues, through the bloodstream or lymphatic system, and establish secondary tumour sites, a phenomenon called metastasis.

Induction of apoptosis in cancer cells has been the gold standard for many anticancer therapeutic approaches. Most phytochemicals that have anticancer properties induce apoptosis in target cells. Apoptosis, morphologically characterised by cell shrinkage and DNA fragmentation, is orchestrated by a series of molecular events that may be induced extrinsically or intrinsically. Apoptosis has largely been defined in the context of activating caspases (cysteine-aspartic proteases) as effectors of the process [2]. In caspase-mediated apoptosis, two classes of caspases, expressed as proenzymes, are involved: initiator caspases (such as caspases 8, 9, and 10) and executioner caspases (such as caspases 3, 6, and 7) [3]. The variety of strategies employed by caspases to kill cells include: (i) inactivating proteins that inhibit apoptosis (e.g., Bcl-2 proteins); (ii) disassembling cell structures such as the nuclear lamina; and (iii) deregulating proteins, leading to either their gain or loss of function [3]. Therefore, caspases or other regulator proteins upstream or downstream of the caspase-dependent apoptotic pathway are potential antitumour molecular targets. Many of the experimental settings where caspase-mediated apoptosis is assessed tend to use indirect evidence for caspase activities, such as the cleavage of poly-(ADP-ribose) polymerase (PARP), DNA fragmentation, release of cytochrome *c* by mitochondria as a proxy for mitochondrial membrane permeabilisation, or procaspase-3 cleavage. 

Notably, apoptosis is not exclusively mediated through caspases, as a growing body of knowledge has demonstrated that apoptosis can also occur independently of caspases [2,4,5,6]. Other effectors have been implicated, including non-caspase proteases (e.g., cathepsins, calpains, granzymes), ex-mitochondrial proteins (e.g., apoptosis-inducing factor (AIF), mitochondrial EndoG), reactive oxygen species (ROS), reactive nitrogen species (RNS), and calcium ions [2]. Some of the morphological or biochemical features (such as DNA fragmentation, mitochondrial membrane permeabilisation, cleavage of Bcl-2 family of proteins, and flipping of phosphatidylserine to the outer leaflet of the plasma membrane) associated with caspase-independent apoptosis are similar to or overlap with those of caspase-dependent apoptosis, which can bias experimental data interpretation towards the latter. As subversion of apoptosis is a key feature of cancer cells, many of the molecules that regulate the process, whether caspase-dependent or -independent, can be targeted therapeutically to treat or prevent cancer. 

**Figure 1:**
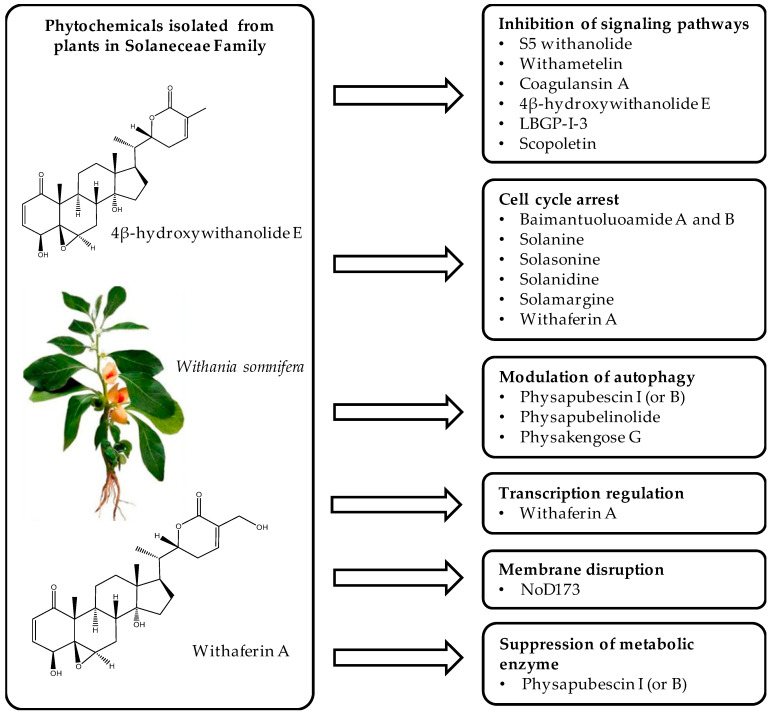
Graphic summary of anticancer mechanisms by phytochemicals isolated from various species in the Solanaceae plant family. A representative species, *Withania somnifera*, is shown with two of its secondary metabolites. The image of *Withania somnifera* was taken from [13], with copyright permission issued by Elsevier and Copyright Clearance Center, under license number 5140970100267.

Plant-derived drugs to treat cancer have been in clinical use for nearly half a century, as exemplified by the vinca alkaloid vincristine, derived from the leaves of *Catharanthus roseus* and approved for clinical use in 1963 [7,8]. However, other drugs not derived from plants are also in clinical use, and these include the most popular cisplatin, a platinum-based transition metal drug [9]. The biggest setbacks with platinum-based compounds are toxicity and drug resistance [10]. To overcome some of the challenges presented by the available drugs, the search for new drugs continues, and nature provides unlimited options through the exploration of plants, particularly their secondary metabolites. Compared to platinum-based drugs, plant metabolites may have lower cytotoxicity [7,11]. Herein, we review the anticancer mechanisms of plant metabolites from the Solanaceae family of plants. The Solanaceae family is very diverse, including trees and herbs that together comprise 3000–4000 species in 90 genera and occupy all terrestrial habitats [12]. Some species have been exploited economically as sources of food (e.g., potato, tomato, pepper), drugs, and ornamentals. Where plants are used for medicinal purposes, understanding their molecular targets is essential to formulating the active compounds into novel plant-derived drugs. Therefore, we shed light on advances that have been made in the last five years to dissect the anticancer mechanisms of compounds isolated from members of the Solanaceae family. These mechanisms are broadly categorised into cell cycle arrest, transcription regulation, modulation of autophagy, inhibition of signalling pathways, suppression of metabolic enzymes, and membrane disruption (Figure 1). 

## 2. Methodology

### 2.1. Search

**Figure 2:**
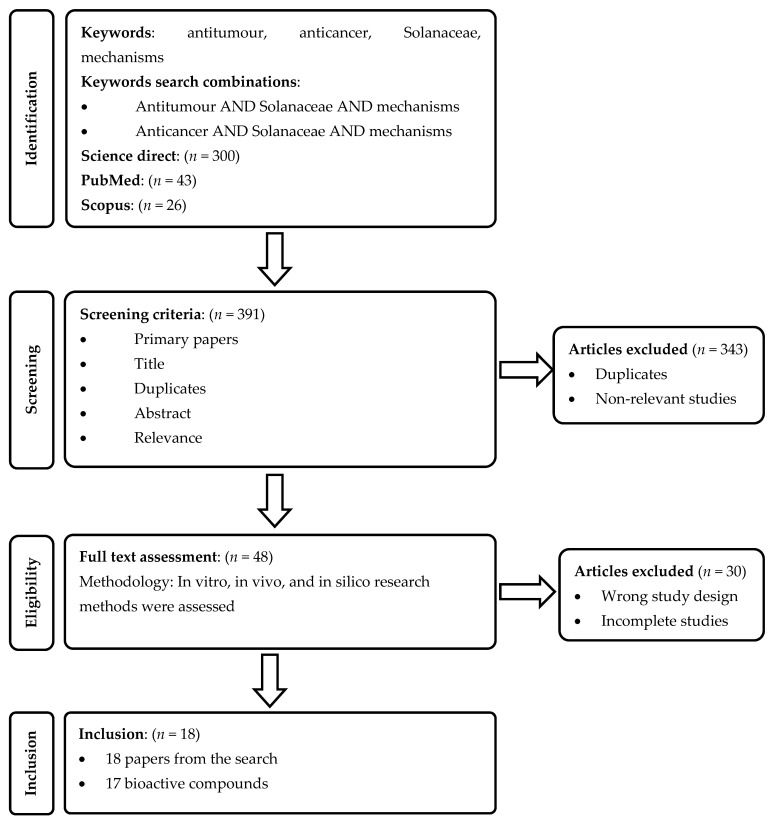
Flow chart summarising the literature search. *n* represents the number of articles.

A literature search was performed using the following databases: ScienceDirect, Web of Science, Scopus and PubMed. We used the keywords ‘Solanaceae AND mechanisms’ in combination with ‘anticancer’ or ‘antitumour’ (Figure 2). The search was restricted to English-language primary journals from January 2016 to April 2021. The database search produced 391 articles that included primary research papers and reviews. We then extracted summary texts including the titles, authors, and abstracts of the studies. 

### 2.2. Screening

The extracted publications included primary research investigating the anticancer or antitumour activities of phytochemicals isolated from plants that belong to the Solanaceae family. Title and abstract reviews were performed to eliminate duplicates and non-applicable articles from the 391 that had been identified, resulting in the elimination of 343 publications.

### 2.3. Eligibility

After critical full-text analysis of the selected studies (48 papers), 30 papers were excluded as they did not explicitly explain the mechanism or where crude plant extracts had been used in the experiments. The criteria for eligibility were the quality of the experimental design and a strong description of the molecular mechanisms involved in the anticancer or antitumour activity of the isolated compounds. In vitro, in vivo, and in silico experimental methods were eligible in the 18 papers that were included, describing 17 bioactive compounds.

## 3. Anticancer Mechanisms

### 3.1. Cell Cycle Arrest

The cell cycle consists of highly orchestrated and distinct phases, monitored through checkpoints that are important for efficient cell proliferation. These phases include G1 (gap 1), S (DNA synthesis), G2 (gap 2), and M (mitosis). To progress efficiently, the cell cycle largely relies on a set of proteins called cyclin-dependent kinases (CDKs) and their activators, cyclins, the levels of which vary in different phases. Successful completion of the cell cycle occurs in response to stimulatory signals including growth factors. Defects or errors, such as DNA mutations or lesions, may lead to cell cycle arrest. This arrest may allow for repair of the errors and completion of the cell cycle in response to stimulatory signals [14]. Cancer cells may arise where errors in the cell cycle surveillance mechanisms are by-passed, leading to the uncontrolled proliferation of cells. Hence, inducing cell cycle arrest is an attractive therapeutic strategy. 

Although cell cycle arrest is one common mechanism through which different phytochemicals exert their antiproliferative effects, the subcellular molecular interactions involved remain largely undefined. To bridge the gap in knowledge and complement in vitro and in vivo assays, in silico and computational analyses (or simply molecular docking studies) have become new and important experimental tools to predict molecular interactions between plant metabolites and cellular factors. Using this technology, Gurushankar et al. [15] studied the potential of newly characterised alkaloids baimantuoluoamide A and baimantuoluoamide B (from plant species such as *Datura metel* L.) as CDK4 inhibitors. CDK4, together with CDK6, is bound and activated by D-type cyclins for the G1-to-S phase transition of the cell cycle [16]. Malfunction of the CDK4–cyclin D1 interaction has been implicated in different types of cancer [17]. Gurushankar and co-workers used molecular docking, molecular dynamics simulations, and the Molecular Mechanics/Generalized Born Surface Area (MM/GBSA) to identify residues at the binding interfaces of baimantuoluoamide A–CDK4 and baimantuoluoamide B–CDK4. Baimantuoluoamide A–CDK4 formed bonds with Val96, Ile12, Leu147, and Val20, which stabilised the complex. Baimantuoluoamide A was also stabilised in the hinge region, where it formed hydrogen bonds with carbonyl groups of Val96 or Leu147. This property was also similar to that displayed by CDK6–p16/p19 complexes. p16 and p19 are inhibitors of CDK4/6 [16]. With respect to baimantuoluoamide B–CDK4 interaction, amino acid residues Phe93, Asp99, and Ile12 were involved, along with Lys35, which is involved in CDK4 inhibition [15]. Both baimantuoluoamide A and baimantuoluoamide B interact with the non-conserved residues of CDK4 in the ATP binding pocket, which are involved in selective CDK4 inhibition. 

Glycoalkaloids from *Solanum* spp., such as solasonine, solanidine, and solamargine, cause significant cell cycle arrest at the S phase, thereby inhibiting cell proliferation, as shown in a comparative study using liver cancer cell lines [18]. In fact, these glycoalkaloids also have anticancer properties against other types of cancer, including skin cancer, breast cancer, colon cancer, and cervical cancer [19,20,21,22,23,24,25]. In a dose–response analysis conducted on liver cancer cell lines, Huh7 and HepG2 cells, higher concentrations of solasonine, solanidine, and solamargine induced significant apoptosis on Huh7 cells [18], suggesting that the use of these compounds may be cancer cell-type-dependent. Furthermore, prolonged exposure (48 vs. 24 h) to the compounds had a more profound antiproliferative effect. In addition to targeting the S phase, solamargine also induced cell cycle arrest at the G2/M phase [18]. However, details on how the glycoalkaloids affect the molecular pathways involved in apoptosis are not clear and need further investigation. One of the important factors in the development of anticancer drugs is selectivity of the effects on cancerous versus healthy cells. A study by Burger et al. [26] revealed that solamargine may not be selective, which may pose as a challenge when attempting to develop it into a drug for cancer. More studies need to be performed to investigate the selectivity of glycoalkaloids.

### 3.2. Transcription Regulation

Modulating transcription is another viable anticancer therapeutic mechanism, achieved by inhibiting oncogenic transcription factors in several ways that include: (i) targeting transcription factors for degradation; (ii) interfering with protein–protein interactions; or (iii) modulating the binding of transcription factors to DNA. Approximately 20% of oncogenes are related to transcription factors [27], making them attractive therapeutic targets. 

One of the most extensively studied oncogenic transcription factors is MYB, which has over 80 target genes. MYB appears to be oncogenic in the bone marrow, colon, and mammary glands, where it is essential for development and/or homeostasis under normal physiologic conditions [28,29,30]. In haematopoietic cell lineages with a progenitor phenotype, levels of MYB are relatively high, but they decline upon differentiation. Notably, the *MYB* gene is amplified and overexpressed in more than 80% of colorectal cancer cases [31,32]. In some breast cancers, expression of *MYB* is enhanced by the presence of another transcription factor, oestrogen receptor alpha (ERα) [33], where a ligand–ERα complex is required to alleviate attenuated expression of *MYB* [34]. Another transcription factor that acts in concert with MYB is the CCAAT/enhancer binding protein (C/EBPβ). The latter belongs to a family of six transcription factors that turn on genes involved in modulating cell proliferation, growth, and differentiation [35]. C/EBPβ displays proliferative and antiproliferative activities in different cell types [36]. One of its interacting partners is the nuclear coactivator p300, an acetyltransferase thought to promote the transcriptional activities of C/EBPβ [37]. 

Given its key role in the development of some cancers, MYB and/or its co-activators are plausible therapeutic targets. This was shown recently for another secondary metabolite, the withanolide withaferin A (WFA) [38]. WFA is isolated from the leaves of *Withania somnifera*, and its anticancer activities were documented in the late 1960s [39]. Several molecular targets of WFA that affect cell proliferation and apoptosis have been unveiled in a number of model cell lines (reviewed by Lee and Choi [40]). The antiproliferative effects of WFA are largely due to arrest of the cell cycle at the G2/M phase, the stage at which a cell has completed DNA synthesis and is committing to mitosis. For example, in HCT116 and SW480 colorectal cell lines, cell cycle arrest at the G2/M phase was mediated through the degradation of Mad2 and Cdc20 proteins, which are required for the activation of the spindle assembly checkpoint [41,42]. Recently, Falkenberg et al. [38] demonstrated that WFA can exert its antitumour activities by inhibiting the transcription factors MYB and C/EBPβ. Their study revealed that WFA could inhibit both MYB and C/EBPβ, although to different extents, with more potency observed on the C/EBPβ transcription factor. They noted that WFA alkylates certain cysteine residues of C/EBPβ, two of which are located in the N-terminus, a region that interacts with the co-activator p300. WFA-induced alkylation is thought to restrict p300 binding to C/EBPβ, and the effect on MYB could be indirect, e.g., through WFA modulating protein–protein interactions at the transcription level. 

### 3.3. Modulating Autophagy

Autophagy is a key process in cellular homeostasis that targets intracellular cargo such as proteins, lipids, organelles, and pathogens for degradation by lysosomes. Autophagy is characterised into three types: macroautophagy, microautophagy, and chaperon-mediated autophagy [43]. Although autophagy may suppress tumour initiation, it is also thought to promote cancer once the disease is established, though this is controversial [44]. Autophagy is mediated through a set of autophagy-related (Atg) proteins that initiate the formation of autophagosomes which ultimately fuse with lysosomes to deliver cargo for degradation by lysosomal hydrolases. Amongst the key regulators of autophagy is the mammalian target of rapamycin complex 1 (mTORC1) pathway, a negative regulator [22]. When mTOR kinase, a component of the mTORC1, is over-activated, cell proliferation is elevated, which may favour the development of tumours [45]. 

Physapubescin B, a withanolide isolated from *Physalis pubescens*, was recently used to treat HCT116 colorectal cancer cells, generating reactive oxygen species (ROS) that resulted in mTORC1 inhibition, thereby activating autophagy, and cell death was also observed [46]. Apoptotic cell death was associated with the cleavage of caspase-3 and poly-(ADP-ribose) polymerase (PARP), which are universally used as cellular markers for apoptosis. Notably, there was activation of the tumour suppressor *p53* gene, the so-called “guardian of the genome”. When physapubescin B was used together with chloroquine, apoptosis was increased. Chloroquine is a drug that collects in lysosomes where it increases the pH and impairs autophagosome–lysosome fusion [47], thus inhibiting autophagy. Although physapubescin B induced autophagy and caused apoptosis, coupling the secondary plant metabolite with an autophagy inhibitor enhanced apoptosis [45]. These results suggested that physapubescin B may have dual effects, where it may either promote cancer survival through autophagy or trigger apoptotic responses to the detriment of the cell. In another equally interesting study, autophagosome formation and apoptosis were concomitantly induced in MDA-MB-231 and MCF-7 human breast cancer cell lines when cells were exposed to physapubenolide, a withanolide extracted from *Physalis angulata* [48]. Using RNA-interference to downregulate some of the key proteins involved in autophagy did not have a significant impact on the viability of cells treated with this phytochemical. Ma and co-workers concluded that the antitumour activity they observed was caused by autophagy as well as apoptosis, as both processes were activated [48]. They suggested that in this case, autophagy was not triggered as a survival mechanism, but to promote elimination of cancer cells. 

Unlike the two metabolites from the *Physalis* genus discussed above, which triggered apoptosis in various cell lines, physakengose G, another new secondary plant metabolite from *Physalis alkekengi* var. *franchetii*, had an effect similar to that of chloroquine when administered to osteosarcoma cell lines U-2 OS and HOS cells [49]. The compound inhibited acidification of lysosomes and rendered them dysfunctional, thereby blocking autophagic degradation and promoting cell death. Inhibition of autophagy was considered potentially therapeutic, at least in a cell line model [49]. Therefore, the diverse effects that some phytochemicals can have on cancer cells mean that data from such studies need to be interpreted with caution.

### 3.4. Inhibition of Signalling Pathways

Genetic alterations have been noted in some of the key signalling pathways that control cell growth and proliferation. These alterations may include gene amplification resulting in the overexpression of genes, point mutations, truncations, or fusions that result in proteins with dysregulated activities. The following are some examples of cell signalling proteins whose altered activities may be tumorigenic: receptor tyrosine kinases, serine/threonine kinases, lipid kinases, and nuclear receptors. Oncogenic mutations can also affect developmental signalling pathways, like the Wnt/β-catenin pathway. Components of these signalling pathways have therefore become targets for the development of anticancer drugs.

Kinase signalling pathways have revealed a wealth of targets for cancer treatment over the years. One class of extensively studied receptor tyrosine kinases (RTKs) are the epidermal growth factor receptors (EGFRs), which stimulate signalling pathways involved in an array of cellular process that promote cell differentiation, proliferation, and migration. EGFRs, like other RTKs, are present as integral membrane proteins that bind ligands and become active upon dimerisation of their subunits. At least seven ligands, including the epidermal growth factor and transforming growth factor alpha, are known [50]. Downstream effectors of the EGFRs include the mitogen-activated protein kinase (MAPK) and phosphoinositide-3 kinase (PI3K, a lipid kinase) pathways, with p38 and AKT serine/threonine-protein kinase as key players, respectively. Some mutations that lead to overexpression of EGFRs have been found in varying proportions in several cancer types, including in 10–30% of breast cancers, 30–50% of glioblastomas, 25–82% of colorectal cancers, and 5–20% of non-small cell lung cancers [51]. The overexpression of EGFR enhances tumour growth progression, as well as blocking apoptosis. Therefore, it is an alternative target for therapy in some cancers. 

In a recent study by Fan and co-workers [52], a novel withanolide simply named S5, isolated from *Physalis pubescens* L., was used to treat human melanoma A375 cells. Cell proliferation was reduced, but there was no change in the expression of caspase 3, PARP, and LC3 proteins, thus ruling out the possibility of apoptosis and autophagy in the observed antiproliferative effect. To dissect the mechanism involved in cell death, analysis of cell cycle phases was carried out, and it emerged that the G2/M phase was arrested, with a reduction in the expression of some of the proteins that regulate the G2/M phase [52]. Further probing into the mechanism revealed that S5 inhibited the expression of phosphorylated EGFR, which ultimately downregulated the expression of p38 in A375 cells. The study by Fan et al. [52] added S5 to the list of compounds that can inhibit receptor tyrosine kinases in cancer treatment (reviewed elsewhere [53,54]). 

In a different approach to study the role of two other withanolides, proteomic profiling was carried out on acute myeloid leukaemia cell lines (Kasumi-1 and P31/FUJ) that had been treated with withametelin and coagulansin A, extracted from *Datura innoxia* and *Withania coagluanse*, respectively [55]. The use of proteomics techniques such as liquid chromatography in conjunction with mass spectrometry (LC-MS) permitted the identification of several proteins that were either up- or downregulated after treatment with these secondary metabolites. Overall, there were reductions in the expression of 33 and 52 proteins in cells treated with coagulansin A and withametelin, respectively [55]. Thereafter, confirmatory functional assays were conducted on a few target proteins, which showed that the compounds exerted their inhibitory effect on the acute myeloid leukaemia cells by downregulating signalling proteins, including those that mediate the PI3K and MAPK pathways. As mentioned earlier, these pathways are involved in RTK (such as EGFR) signalling. However, the study did not definitively implicate receptor tyrosine kinases in the anticancer mechanism. An analysis of apoptotic markers in functional assays showed induced cleavage of PARP and caspases 3, 8, and 9 in a dose-dependent manner. 

Other potential secondary metabolites acting on the AKT signalling pathway include 4β-hydroxywithanolide E (a metabolite from *Physalis peruviana*) and WFA. The role of WFA interfering with transcription has already been discussed. In addition, WFA may have a role pertaining to the inhibition of AKT signalling, as determined in a comparative study where breast cancer MDA-MB-231 cells were subjected to increasing doses of either 4β-hydroxywithanolide E or WFA [56]. Comparative analysis of the effect that the two metabolites had on the cells showed that, although both could suppress AKT signalling, very high doses of WFA were required to effectively inhibit the pathway. Although the antiproliferative effect of 4β-hydroxywithanolide E was mainly due to suppression of AKT signalling, WFA blocked the G2/M phase of the cell cycle. WFA also caused apoptosis by inhibiting the molecular chaperone heat shock protein 90 (Hsp90), consequently depleting its binding partner Raf-1, a serine/threonine kinase whose stability and cellular localisation depend on Hsp90 [57,58]. Several studies have demonstrated that Hsp90 could be a druggable antitumour target (reviewed in [57,59,60]).

Seemingly, 4β-hydroxywithanolide E may have broad-spectrum applications as it can also target components of another signalling pathway, the Wnt/β-catenin signalling pathway, in colorectal cancer [61]. In the pathway, extracellular Wnt ligands engage their membrane frizzled receptors and LRP5/6 co-receptors [62]. The intracellular dishevelled (DVL) protein is activated, and it causes aggregation, to the frizzled receptor, of a destruction complex made up of four components: glycogen synthase kinase 3β (GSK3β), Axin, adenomatous polyposis coli (APC), and casein kinase 1α (CK1α) [63]. Aggregation and phosphorylation of GSK3β favour an increase in the amount of free β-catenin in the cell, allowing it to translocate to the nucleus. β-catenin acts as a transcription co-factor regulating the expression of target genes for proteins such as cyclin D1, c-Myc, and survivin [64]. When Wnt ligands are not receptor-bound (i.e., in an “off” mode), GSK3β in the destruction complex phosphorylates β-catenin, thereby priming it for ubiquitination and subsequent proteasomal degradation. In the absence of β-catenin, target genes are not transcribed. Therefore, targeting β-catenin where the Wnt signalling pathway is upregulated, such as in colorectal cancer, may be key to the development of novel treatment options. Exposure of the colorectal cancer cell lines HT29, HCT116, and SW480 to 4β-hydroxywithanolide E caused a decline in the expression of cyclin D1, c-Myc, and Axin 2 [61]. When RNA-interference was used to downregulate β-catenin mRNA, the effect of 4β-hydroxywithanolide E was reduced, suggesting that the compound may be, at least in part, acting at the transcriptional level, which may explain the reduction in the expression of target genes for the proteins cyclin D1, Axin2, Myc-1, and survivin. Similar observations of reduced gene expression were made during in vivo studies where HCT116 xenografts were used. 

The MAPK signalling cascades are emerging as important players in cancer pathobiology. Earlier, we discussed MAPK signalling in the context of the RTK signal transduction pathway. At least three families of MAPK cascades are known: (i) extracellular signal-regulated kinases (ERK), (ii) c-jun N-terminal kinase (JNK), and (iii) p38 kinases [65]. JNK and p38 MAPKs are normally co-activated during overexpression of MAP kinase kinases (MAP3K) [66]. Through an extrinsic or intrinsic stimulus, these kinases phosphorylate their specific substrates at threonine and serine residues, leading to activation or deactivation of proteins that control apoptosis. JNK modulates the activity of the tumour suppressor gene *p53* and acts as an antagonist to the anti-apoptosis proteins MCL-1 and Bcl-XL [67,68]. p38 kinase is involved in the regulation of G1 cell cycle arrest through CDC25 phosphatase inhibition. CDC25 phosphatases regulate cyclin-dependent kinases, and their overexpression in cancer cells leads to increased cell proliferation and poor diagnosis [69]. Recently, one study showed that some plant polysaccharides can induce apoptosis through the JNK and p38 MAPK signal transduction pathways [70]. The researchers isolated a polysaccharide called arabinogalactan (LBGP-I-3) from *Lycium barbarum* fruits and then used it to treat the human breast cancer cell line MCF-7. LBGP-I-3, a highly branching polysaccharide composed of arabinose and galactose, was found to upregulate p-JNK and p-p38 and downregulate p-ERK 1/2. Additionally, cell cycle arrest was observed at G0/G1. 

In a study by Wen and co-workers [71], α-solanine promoted the degradation of vascular endothelial growth factor (VEGF), and promoted the expression of E-cadherin. Whilst E-cadherin reduces migration of cancer cells by modulating cell adhesion, VEGF interacts with RTKs at the cell surface and can stimulate several signalling pathways, including (i) the Ras/MAPK pathway that controls cell proliferation and gene expression; (ii) the FAK/paxillin pathway responsible for rearrangement of the cytoskeleton; (iii) the PI3K/AKT pathway that modulates cell survival; and (iv) the PLC pathway regulating vascular permeability [72]. Under hypoxic conditions, VEGF levels increase, and E-cadherin is reduced in human pancreatic cells; both effects are reversed by the addition of α-solanine [71]. This indicates that α-solanine can form part of a potential strategy to address pancreatic cancer, since it could prevent angiogenic activity and migration of pancreatic cancer cells. The investigation also showed that α-solanine resulted in decreased expression of HIF-1α, which regulates the expression of a series of genes involved in cell survival, angiogenesis, migration, and invasion [71]. According to other studies, inhibitors of HIF-1α represent good candidates for new anticancer drugs [73,74,75]. Results from Wen et al. [71] suggested that α-solanine inhibited VEGF through a pathway that involves HIF-1α, the ERK1/2-HIF-1α pathway. It also inhibited another VEGF promoter, STAT3, which is an important transcription activator during angiogenesis [71,76]. Therefore, the effect of α-solanine on VEGF levels may be through both HIF-1α and p-STAT3. STAT3 inhibitors have also been shown to decrease HIF-1α expression. A number of research groups have shown that some drugs have similar effects on different types of cancer cells [77,78,79]. Therefore, α-solanine may be explored as an anticancer drug for various cancers. 

Another metabolite studied using an in silico approach is scopoletin from *Nicotia glauca.* A molecular docking approach using the LeadIT FlexX scoring functions demonstrated that scopoletin has strong binding affinity and ligand efficiency with VEGFA, ERK-1, and fibroblast growth factor 2 (FGF2) involved in signalling [80].

### 3.5. Suppression of Metabolic Enzymes

A common phenomenon that allows cancer cells to support their abnormal behaviours is reprogramming of metabolic pathways. Cancer cells have high energy requirements to cope with their elevated metabolism. Therefore, targeting some of the key enzymes that are involved in ATP generation may be a viable approach to shutting down the growth and proliferation of tumours. One possible target is the mitochondrial glutaminase, which is required for the conversion of glutamine to glutamate in a process called glutaminolysis [81]. Glutamate can then be used to drive the tricarboxylic acid cycle or Krebs cycle A, to meet the high energy demands of cells. In mammals, two types of glutaminase are expressed: the kidney type and the liver type. The latter is thought to be significant in various cancers. In a search for phytochemicals that could potentially inhibit the kidney-type glutaminase, Yang et al. [82] conducted a molecular docking study and found that physapubescin I (also known as physapubescin B [45]), derived from *Physalis pubescens* L., could occupy the active site of the enzyme, thereby blocking the entry of substrate into the binding site. Confirmatory functional assays were carried out and established that: (i) siRNA-mediated depletion of kidney-type glutaminase abated the inhibitory effect of physapubescin I in human pancreatic cancer cells SW1990; and (ii) supplementation of physapubescin-I-treated cells with alpha-ketoglutarate abolished the inhibition. Lastly, in vivo studies with SW1990 xenograft mice further demonstrated the antitumorigenic effects of physapubescin I, without obvious pathological changes in the liver, spleen, kidneys, and pancreas. The study marked an important addition to the repertoire of secondary metabolites from the genus *Physalis* that display anticancer properties. 

### 3.6. Membrane Disruption

In the development of cancer treatments, it is important to develop anticancer compounds that have selective cytotoxic effects on cancer cells only. The overexpression and exposure of negatively charged phospholipids like phosphatidylserine (PS) in the outer leaflet of the plasma membrane is a common property of various types of cancer cells [83]. The exposed negatively charged phospholipids contribute to the overall negative charge of the plasma membrane of cancer cells. This property contributes to the anticancer specificity of some plant peptides [84] in that their activities are related to their ability to interact with negatively charged plasma membrane phospholipids and phosphatidic acid [85]. The three-dimensional orientation of the different plant peptides is characterised by a grip-shaped cationic binding pocket that allows for ionic and hydrogen bond interactions with the cancer cell plasma membrane [85]. The plant peptide–plasma membrane interaction is mediated through a carpet-like model or detergent-like conformation that leads to compromised plasma membrane integrity and cell membranolytic activity [86]. 

Some plant peptides can cause apoptosis by targeting the mitochondrial membrane. They permeabilise the membrane, collapsing the mitochondrial membrane potential and leading to release of cytochrome *c* and the activation of caspases [85]. In a recent investigation, a plant peptide isolated from *Nicotiana occidentalis*, *Nicotiana occidentalis* defensin (NoD173), showed anticancer activity on human cervical adenocarcinoma (HeLa) and malignant melanoma (MM170) cell lines [86]. It also halted cell proliferation on solid B16-F1 skin melanoma in a mouse model. NoD173 has a net positive charge of +8, and further increasing its cationicity by substituting glutamine at position 22 with lysine (NoD173_Q22K_) was shown to increase its cytotoxic effect twofold. The peptide showed selective binding to phosphatidylinositol 4,5-bisphosphate (PIP2) in a protein–lipid overlay assay. Crystallisation and structure determination of NoD173 revealed the presence of a phospholipid cationic binding pocket functional unit on the peptide. A similar cationic binding pocket was also shown to be present in the anticancer plant peptide NAD1 isolated from *Nicotiana alata* and *Petunia hybrida* plants from the Solanaceae family [87,88]. 

## 4. Conclusions

In this review, we provided an update on advances that have been made to understand how different plant metabolites from the Solanaceae family (summarised in Table 1) exert antiproliferative effects on cancer cells. Most of the studies that we assessed employed in vitro assays and, to a small extent, in vivo assays. In some cases, in silico studies were used alone or to complement in vitro or in vivo functional assays. Through molecular docking studies, the amino acid residues most important for stabilising host protein–metabolite interactions could be identified. This may be important in the context of single-nucleotide polymorphisms that could change the residues and affect the stability of the complexes. Whilst there were several molecular targets for various compounds, induction of apoptosis was the ultimate outcome. From the studies we evaluated, it emerged that a single metabolite may have several molecular targets. Multifunctional phytochemicals such as WFA thus represent versatile compounds, possibly demonstrating superiority over other compounds and suggesting that they are good drug candidates for diverse cancer types. Also of note is the fact that the genetic makeup of a cell can dictate how it responds to a compound. For example, HCT116 cells that express the *p53* gene are relatively more responsive to treatment by physapubescin B compared to *p53* negative cells. Given that the studies presented in this review were predominantly conducted on cell lines, further research on animal models is required, leading to clinical studies. 

## Figures and Tables

**Table 1:** Summary of plant metabolites and their mechanisms of action.

Phytochemical and Plant Sources	Chemical Structures	Experimental Model	Mechanisms of Action	Refs
Baimantuoluoamide A *Datura metel* L.	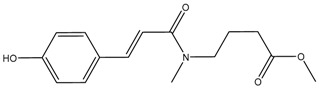	Molecular docking and molecular dynamics simulations Human CDK4 (PDB entries 2W9Z and 1GII)	Cell cycle arrest CDK4 inhibition	[15]
Baimantuoluoamide B *Datura metel* L.	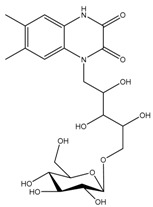			
Coagulansin A *Withania coagluanse*	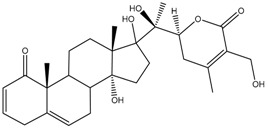	Acute myeloid leukaemia (AML) cell lines, i.e., HL60, Kasumi-1, and P31/FUJ	Inhibition of signalling pathway Downregulates proteins in PI3K and MAPK pathways Showed induction of cleavage of PARP and caspases 3, 8, and 9	[55]
Hydroxywithanolide E (or 4β-hydroxywithanolide) *Physalis peruviana, Withania somnifera* L.	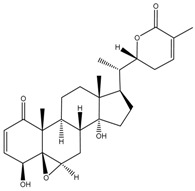	Human colorectal carcinoma cell line, i.e., HCT116 and HT-29 Human breast cell line, i.e., MDA-MB-231 and MDA-MB-468	Inhibition of signalling pathway Inhibition of AKT signalling Downregulation of Cyclin D1, c-Myc, and Auxin	[56,61]
*L. barbarum* fruit arabinogalactan (LBGP-I-3) *Lycium barbarum*	Highly branched polysaccharide composed of arabinose (48.15%) and galactose (44.44%)	Human breast cancer cells (MCF-7)	Inhibition of signalling pathway Upregulation of c-JNK and p38 Downregulation of p-ERK 1/2 Cell cycle arrest At G0/G1 phase	[70]
*Nicotiana occidentalis* defensin 173 (NoD173) *Nicotiana occidentalis*	NoD173 is a peptide molecule whose three-dimensional structure has been explained through Nuclear Magnetic Spectroscopic methods as well as Protein-Crystallographic methods.	Solid B16-F1 mouse melanoma	Membrane disruption Interaction with PIP2	[86]
Physapubescin I (or B) *Physalis pubescens* L.	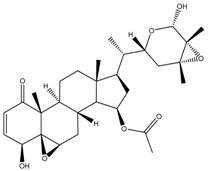	Molecular docking Human KGA (PDB entry 3VP1) Human colorectal carcinoma cell line (HCT116) Human cervical cancer cell line (HeLa cells) Human pancreatic cancer cell line (SW1990)	Modulation of autophagy Suppression of metabolic enzyme Suppress glutaminase	[46,82]
Physakengose G *Physalis alkekengi* var. *Franchetii*	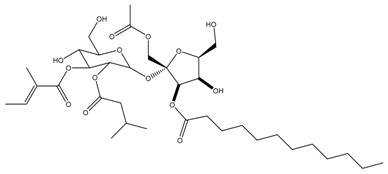	Human osteosarcoma cell lines, i.e., U-OS and HOS cells	Modulation of autophagy Apoptosis due to EGFR signalling	[49]
Physapubenolide *Physalis angulate*	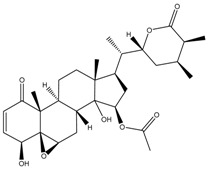	Human breast cancer cell lines, i.e., MDA-MB-23 and MCF-7 cells	Modulation of autophagy	[48]
S5 withanolide *Physalis pubescens* L.	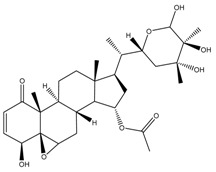	Human melanoma cell line (A375)	Cell cycle arrest At G2/M phase Inhibition of signalling pathway Inhibits EGFR phosphorylation	[52]
Scopoletin *Nicotiana glauca*	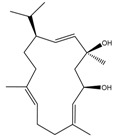	Human colorectal tumour xenograft model Molecular docking (LeadIT FlexX method) Angiogenic mitogens, i.e., ERK1/2, VEGF-A, and FGF-2	Inhibition of signalling pathway Binds VEGFA, ERK-1, and FGF2	[80]
Solamargine *Solanum aculeastrum* Dunal, *S. melongena, S. nigrum*	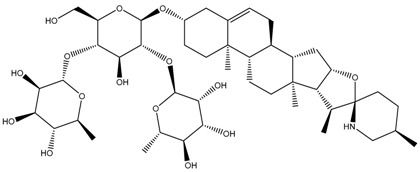	Human liver cancer cell lines, i.e., HepG2 and Huh-7 cells Human neuroblastoma cell line (SH-SY5Y)	Cell cycle arrest At S and G2/M phases	[18,25,26]
Solanine (or α-solanine) *Solanum melongena*	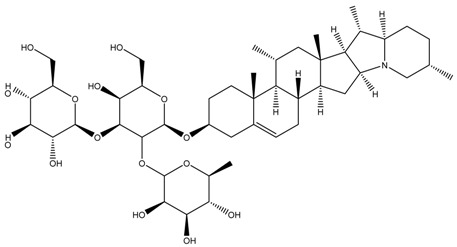	Human pancreatic cancer cell lines	Inhibition of signalling pathway Inhibition of VEGF through degradation Inhibition of STAT3 pathway Increases expression of HIF-1α and E-cadherin Cell cycle arrest At S phase	[18,71]
Solanidine *S. nigrum*	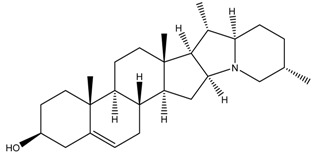	Human lung adenocarcinoma cell line (A549) A549 CAM xenograft BALB/c mouse model	Cell cycle arrest At S and G2/M phase	[23]
Solasonine *S. nigrum*	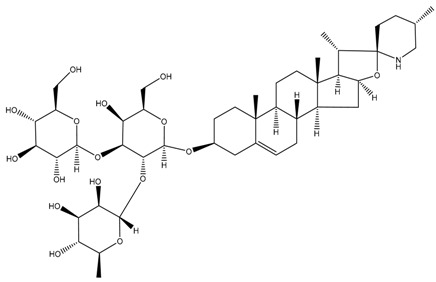	Human breast cancer cell line (Bcap-37) Liver cancer cell lines, i.e., Huh7 and HepG2	Cell cycle arrest At S phase	[25]
Withaferin A *Withania somnifera*	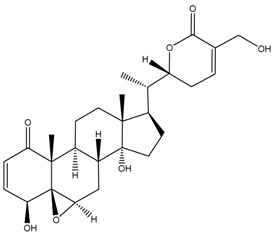	Chicken myeloid cell lines (HD11-C3-GFP1 Myb reporter cell line) Human Myeloid leukaemia cell line (HL60) Quail Japanese fibrosarcoma (QT6) Mouse preadipocyte cell line (3T3-L1)	Cell cycle arrest At G2/M phase Transcription regulation Inhibition of transcription factors MYB and C/EBPβ	[38,56]
Withametelin *Datura innoxia*	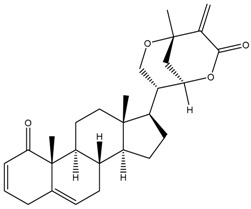	Human acute myeloid leukaemia cell lines, i.e., HL60, Kasumi-1 and P31/FUJ	Inhibition of signalling pathway Downregulates proteins in PI3K and MAPK pathways Induction of cleavage of PARP and caspases 3, 8 and 9	[55]
